# Impact of Epithelial–Mesenchymal Immunophenotype on Local Aggressiveness in Papillary Thyroid Carcinoma Invading the Airway

**DOI:** 10.3390/jcm10194351

**Published:** 2021-09-24

**Authors:** Martina Mandarano, Marco Andolfi, Renato Colella, Massimo Monacelli, Andrea Polistena, Sonia Moretti, Guido Bellezza, Efisio Puxeddu, Alessandro Sanguinetti, Angelo Sidoni, Nicola Avenia, Francesco Puma, Jacopo Vannucci

**Affiliations:** 1 Section of Anatomic Pathology and Histology, Department of Medicine and Surgery, University of Perugia, 06132 Perugia, Italy; martina.mandarano@unipg.it (M.M.); renato.colella75@gmail.com (R.C.); guido.bellezza@unipg.it (G.B.); angelo.sidoni@unipg.it (A.S.); 2 Thoracic Surgery Unit, AOU Ospedali Riuniti di Ancona, 60126 Ancona, Italy; marcoandolfi@hotmail.com; 3 Thoracic Surgery Unit, University of Perugia Medical School, 06132 Perugia, Italy; massimo.monacelli@gmail.com (M.M.); francesco.puma@unipg.it (F.P.); 4 Department of Surgery “Pietro Valdoni”, Policlinico Umberto I, University of Rome “Sapienza”, 00161 Rome, Italy; andrea.polistena@uniroma1.it; 5 Internal Medicine and Endocrine and Metabolic Sciences Unit, University of Perugia Medical School, 06132 Perugia, Italy; sonia.moretti@unipg.it (S.M.); efisio.puxeddu@unipg.it (E.P.); 6 General and Specialized Surgery, University of Perugia Medical School, Santa Maria Hospital, 05100 Terni, Italy; a.sanguinetti@aospterni.it (A.S.); nicola.avenia@unipg.it (N.A.); 7 Thoracic Surgery and Lung Transplantation Unit, Policlinico Umberto I, University of Rome “Sapienza”, 00161 Rome, Italy

**Keywords:** thyroid cancer, papillary thyroid carcinoma, poorly differentiated thyroid carcinoma, airway, trachea, neck, endocrine tumours, biomarkers, immunohistochemistry

## Abstract

Primary thyroid tumours show different levels of aggressiveness, from indolent to rapidly growing infiltrating malignancies. The most effective therapeutic option is surgery when radical resection is feasible. Biomarkers of aggressiveness may help in scheduling extended resections such as airway infiltration, avoiding a non-radical approach. The aim of the study is to evaluate the prognostic role of E-cadherin, N-cadherin, Aryl hydrocarbon receptor (AhR), and CD147 in different biological behaviours. Fifty-five samples from three groups of thyroid carcinomas were stained: papillary thyroid carcinomas (PTCs) infiltrating the airway (PTC-A), papillary intra-thyroid carcinomas (PTC-B) and poorly differentiated or anaplastic thyroid carcinomas (PDTC/ATC). High expressions of N-cadherin and AhR were associated with higher locoregional tumour aggressiveness (*p* = 0.005 and *p* < 0.001 respectively); PDTC/ATC more frequently showed a high expression of CD147 (*p* = 0.011), and a trend of lower expression of E-cadherin was registered in more aggressive neoplasms. Moreover, high levels of AhR were found with recurrent/persistent diseases (*p* = 0.031), particularly when tumours showed a concomitant high N-cadherin expression (*p* = 0.043). The study suggests that knowing in advance onco-biological factors with a potential role to discriminate between different subsets of patients could help the decision-making process, providing a more solid therapeutic indication and an increased expectation for radical surgery.

## 1. Introduction

Thyroid cancer is the most common endocrine malignancy. This disease varies from indolent tumour to highly aggressive disease [1]. Indeed, although early-stage well-differentiated thyroid cancer (DTC) has a good prognosis after surgery [2,3,4], those tumours invading surrounding tissues (extra-thyroid extension) show an increased persistence/recurrence of disease and decreased survival [5,6,7].

Airway invasion is found in approximately 6% of the total thyroid tumours and can determine different clinical conditions. These patients can show normal breathing or severe obstruction in the most advanced disease progression [5,6]. In such conditions, the ideal surgical treatment is still a matter of debate. Many approaches, single or combined multi-modality treatment, have been described with disputed results [8]. In particular, concerning the airway invasion, the shaving-off of the tumour from the airway or tracheal window resection is performed in the case of superficial invasion, while segmental resection is preferred when the airway invasion is deeper in the laryngo-tracheal wall [4]. However, some authors underline the weakness of the shaving and tracheal window [4,8], emphasising that segmental resection with end-to-end anastomosis should be preferred even in limited infiltration to reduce risk for recurrence and airway damage. Moreover, other conventional therapies, such as radioactive iodine, thyroid hormone therapy and chemotherapy, have been developed and performed with growing trends and promising perspectives [9,10,11,12].

Despite the progress obtained in the recent past in terms of diagnosis, staging and therapeutic options [13,14,15], and in view of the lack of markers to predict the oncological outcome, there is still a need for biological, genetic and immunohistochemical (IHC) indicators to develop a more effectively tailored approach. Based on the current knowledge regarding potential biomarkers of tumour aggressiveness, Aryl hydrocarbon receptor (AhR), N-cadherin, E-cadherin and CD147 were selected to be analysed in thyroid carcinomas for the following reasons. AhR has been widely analysed and seems to be associated with tumour genesis and different disease progression phases [16]. In a poorly differentiated thyroid carcinoma (PDTC) cell line, kynurenine-driven activation of AhR induced epithelial–mesenchymal transition (EMT) involving cadherins [17]. The transmembrane glycoprotein CD147 is known to facilitate tumour cell migration and invasion in several cancers [18]; matrix metalloproteinases (MMPs) seem to be activated [19], and CD147 possibly promotes the mesenchymal phenotype with cadherin expression variations [20,21]. A high expression of CD147 was described in DTC [22], with an emphasis on lymph node metastasis and tumour invasion [23].

The aim of the study is to compare the immunophenotypic characteristics of thyroid tumours invading the airway and complete intra-thyroid tumours in order to generate a hypothesis for possible new markers of local aggressiveness and to determine how the aggressive tumour attitude could be predicted.

## 2. Material and Methods

A comparative retrospective observational study on the expression of E-cadherin, N-cadherin, AhR and CD147 was performed on a series of patients undergoing surgery (2010–2017) for papillary thyroid cancer. The presence of accurate pathological reports, information regarding nodal status at the diagnosis and/or data regarding the recurrence and/or persistence of disease were used to select the patients eligible for the study. Subsequently, 3 groups of patients were considered: patients with papillary thyroid carcinoma (PTC) invading larynx and trachea undergoing total thyroidectomy with segmental resection of the airway (PTC-A group); patients with completely intrathyroidal PTC (PTC-B group); and patients with PDTC or anaplastic thyroid carcinomas (ATC) (PDTC/ATC group), which served as a control group for more locally aggressive neoplasms. The study was approved by the local ethics committee (N. 23665/10/AV of 26 January 2010).

### 2.1. Study Population

Patients were included the analysis if they were over 18 years of age; histologically diagnosed for PTC, PDTC or ATC; and followed up by the Internal Medicine and Endocrine and Metabolic Sciences Unit, University of Perugia. Patients with follicular and medullary tumours were excluded. Medical history, endoscopic findings, pathological reports, work for the assessment of possible resection with curative intent, indication to airway resection or other therapeutic options, histo-pathological aspects, immunophenotypic profile and adjuvant therapies were evaluated. At admission, all the patients underwent routinary tests for an appropriate preoperative assessment.

### 2.2. Surgical Procedures

Based on the different disease characteristics, two different procedures of surgical resection were carried out: total thyroidectomy with central compartment lymphectomy and latero-cervical lymphectomy (in the case of positive ultrasound (US) lymph node involvement) was performed for all cyto-histologically proven PTCs or early-stage PDTCs. In the cases of tumours infiltrating the airway, total thyroidectomy + lymphectomy was associated with resection and end-to-end anastomosis of the airway according to Grillo’s technique [24]. Diagnosed ATCs were excluded from radical surgery due to stage and local conditions but were submitted to palliative procedures (endoscopy or tracheotomy) and alternative treatment.

### 2.3. Histopathological and Immunohistochemical Determinations

The surgical specimens were fixed in 4% buffered formalin and paraffin embedded (FFPE). Four-micrometer-thick sections were used to obtain both the haematoxylin and eosin (H&E) (Leica ST5020 Multistainer (Leica Biosystems, Nußloch, Germany)), using the ST Infinity H&E Staining System kit (Leica Biosystems, Richmond, IL, USA), and the IHC stains (BOND-III fully automated immunohistochemistry stainer (LeicaBiosystems, Nußloch, Germany)) and peroxidase immunoenzymatic reaction with development in diaminobenzidine, including proper positive and negative controls.

The tumour areas and the tumour histotypes, according to the World Health Organization’s classification of endocrine organ tumours, 2017, 4th Ed.—in force at the time of the study—were identified on H&E slides, allowing the identification of 3 groups of tumours: PTC, PDTC and ATC. Among these last two groups, tumours without a pure histotype were not considered for the analyses. Moreover, the presence of extra-thyroid infiltration was assessed on H&E slides.

The IHC stains were set up using antibodies against E-cadherin (Leica Biosystems, Newcastle Upon Tyne, United Kingdom, Cat# PA0387, RRID:AB_442084, ready-to-use), N-cadherin (ThermoFisher Scientific, Rockford, IL, USA, Cat# 33-3900, RRID:AB_2313779, dilution 1:150), AhR (ThermoFisher Scientific, Rockford, IL, USA, Cat# MA1-514, RRID: AB_2273723, dilution 1: 250) and CD147 (ThermoFisher Scientific, Rockford, IL, USA, Cat# MA1-19201, RRID: AB_1071293, dilution 1:100). Two trained pathologists (M.Ma. and R.C.) separately performed a semi-quantitative evaluation of the immunostains, using a H-Score—as previously reported for AhR [17]—which was the result of the intensity of the staining (0 = absent, 1 = mild, 2 = moderate, 3 = intense) multiplied by the percentage of labelled tumour cells. Two other expert pathologists (G.B. and A.Si.) convened to compare discordant cases in order to assign a definitive H-score. Afterwards, the low and the high classes of expression were obtained, depending on whether the H-score was lower or higher than the internally validated cut-offs—laboratory developed test (LDT)—for each protein investigated (Table 1). As regards E-cadherin, the tumour belonged to the normal expression group when the H-score was >200 and to the lost/low group when the H-score was ≤200.

### 2.4. Statistical Analysis

Descriptive statistics were used for the analysis of immunomarker expressions. Linear correlations between the expressions of the immunomarkers were analysed using the Pearson correlation coefficient. Categorical variables were presented as frequencies with row and column percentages and compared between the groups using chi-square test or Fisher’s exact test as appropriate. Values of *p* < 0.05 were assumed as significant.

## 3. Results

### 3.1. Histopathological Findings

The H&E slides examination identified 8 (15%) PTC-A out of 55 enrolled cases, 27 (49%) PTC-B and 20 (36%) PDTC/ATC. Moreover, H&E enabled the choice of the appropriate tumour areas for the following IHC analysis. Among the PDTC/ATC (13/7), 4/13 PDTC and 1/7 ATC showed more differentiated tumour areas and were therefore not considered for further analysis between immunomarker expressions and clinical–pathological characteristics of the tumours. The clinical characteristics of the PTC-A patients are summarised in Table 2.

### 3.2. Immunohistochemical Analyses

**Figure 1:**
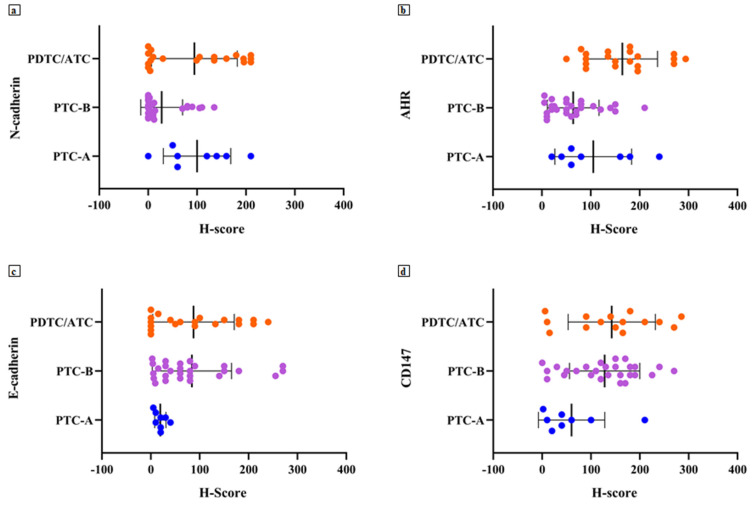
Scatter plots of H-score distribution for N-cadherin (**a**), AhR (**b**), E-cadherin (**c**) and CD147 (**d**) according to tumour groups analysed.

The obtained H-score values showed a mean value of 62.49 ± 73.49 (range 0–210) for N-cadherin, 106.6 ± 78.19 (range 5–294) for AhR, 121.5 ± 80.34 (range 0–285) for CD147 and 75.80 ± 78.66 (range 0–270) for E-cadherin (Figure 1).

**Figure 2:**
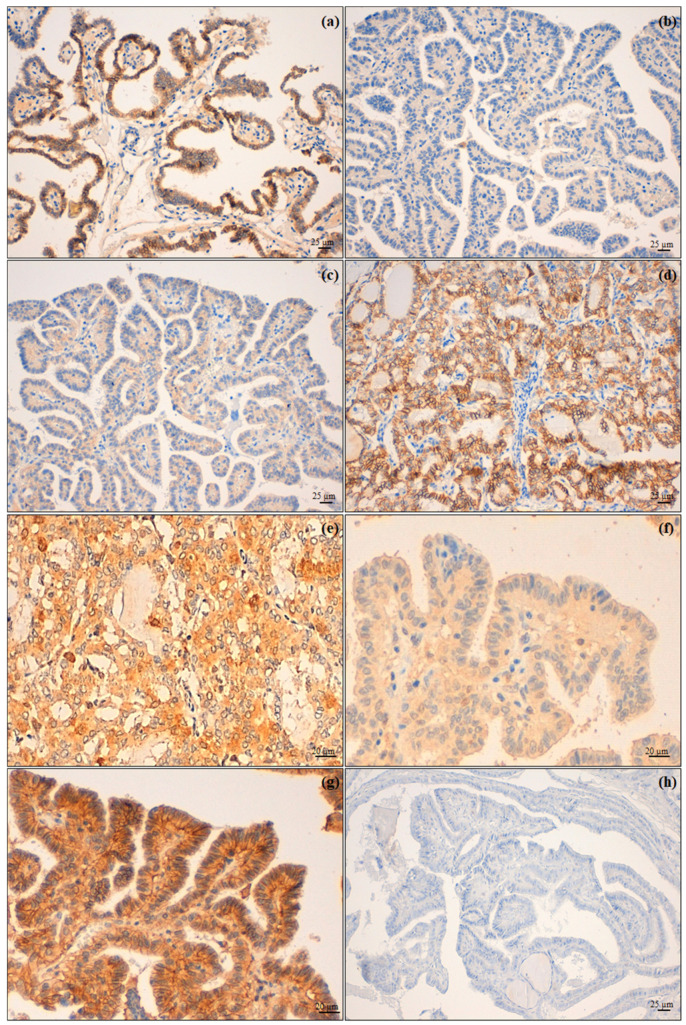
Biomarker immunohistochemical expression. (**a**) Membranous high expression of N-cadherin on tumour cells of PTC infiltrating the airway (PTC-A) compared with (**b**) an intrathyroid PTC (PTC-B); (**c**) PTC-A presented a low expression of E-cadherin and (**d**) PTC-B (follicular variant) with retained expression of this molecule; (**e**) PTC-A showing a high expression of AhR in contrast with (**f**), which shows low expression of the same immunomarker in a PTC-B; (**g**) CD147 high expression of PTC-B and (**h**) absent expression in PTC-A. Original magnification: (**a**–**d**,**h**) 200×; (**e**–**g**) 400×.

Regarding N-cadherin expression, 30 cases (55%) belonged to the low expression class for this protein; 7 (87%) PTC-A and 11 (55%) PDTC/ATC showed high N-cadherin expression. Therefore, the higher the tumour aggressiveness, the higher the N-cadherin expression (*p* = 0.005; Table 3, Figure 2).

Thirty-two tumours (58%) showed a high expression of AhR, and 19 of these (95%) belonged to the PDTC/ATC group (*p* < 0.001; Table 3). A higher expression of AhR was found in PTC infiltrating the airway compared to intrathyroid tumours (50% of PTC-A vs. 33% of PTC-B; Figure 2).

CD147 presented a high expression in 43 cases (78% of the total caseload), with 23 (85%) in the PTC-B group and 17 in the PDTC/ATC group (85%) (*p* = 0.011; Table 3, Figure 2).

Most tumours (49, 89%) presented a lost/low expression of the E-cadherin; all 8 PTC-A cases (100%) presented a lower expression of E-cadherin, while 3 out of 20 PDTC/ATC cases (15%) retained its expression (Table 4; Figure 2).

### 3.3. Correlations between Immunomarkers

**Figure 3:**
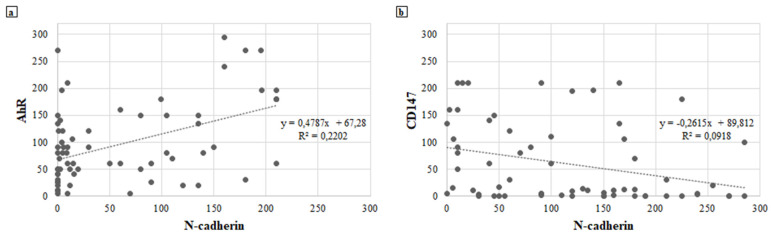
(**a**) Positive relationship between the expression of N-cadherin and AhR. (**b**) Negative relationship between the expression of N-cadherin and CD147.

A moderate positive correlation between the expression of N-cadherin and AhR is reported if H-score values are considered as continuous variables; i.e., the increase in N-cadherin expression follows the increase in AhR and vice versa (ρ = 0.540; *p* < 0.001; Figure 3a).

Furthermore, the higher the expression of N-cadherin, the lower the expression of CD147 (ρ = −0.426; *p* = 0.002; Figure 3b). An inverse correlation trend was observed between the other tested immunomarkers. When the expression of CD147 increases, both E-cadherin (ρ = −0.112; *p* = 0.438) and AhR (ρ = −0.256; *p* = 0.073) decrease (Figure S1a,b, respectively). On the other hand, there was a positive correlation between the expressions of AhR and E-cadherin, but this was not statistically significant (ρ = 0.124; *p* = 0.366; Figure S2).

### 3.4. Immunomarker Expression and Clinical–Pathological Associations

The results regarding the analysis of the associations between the IHC expression of N-cadherin, AhR, CD147 or E-cadherin and histotype, multifocality of the disease, nodal status and tumour recurrence/persistence are shown in Table 5. Some data were not available when the series review was performed.

In particular, high expression of AhR was statistically correlated with a more aggressive histotype of thyroid cancer: 14 PDTC/ATC (93%) showed a high AhR expression (*p* < 0.001, Table 5). Moreover, the cases with recurrence/persistence of disease more frequently expressed high levels of AhR (18 cases, 72%; *p* = 0.031, Table 5). N-cadherin was more frequently observed in PDTC/ATC histotype (9 cases, 60%, not significant, *p* = 0.193, Table 5) and in tumours presenting a recurrence/persistence of the disease (15 cases, 60%, not significant, *p* = 0.182, Table 5). CD147 and E-cadherin did not show a statistically significant association with the clinical–pathological parameters considered (Table 5).

Concomitant high levels of AhR and N-cadherin (9 PDTC/ATC cases, 90%) seemed to be associated with a more aggressive tumoural behaviour (*p* = 0.002; Table 6), as already shown in cases of the expression of both AhR (statistically significant, *p* < 0.001) and N-cadherin (showing just a trend, *p* = 0.193) when singularly analysed (Table 5). Moreover, 12 cases (75%) with high AhR and N-cadherin showed a recurrence/persistence of the disease (*p* = 0.043; Table 6).

When immunomarker expressions were evaluated in PTC cases in view of the presence (PTC-A) or absence (PTC-B) of the infiltration of the airway, high N-cadherin was statistically associated with the tumours presenting airway infiltration (*p* = 0.003; Table 7). CD147 expression was lower in PTC-A (5 cases, 56%; *p* = 0.015, Table 7), supporting the finding of an inverse correlation between this protein and N-cadherin (*p* = 0.002; Figure 3b).

## 4. Discussion

Thyroid carcinoma is a very heterogeneous disease with a different prognosis based mainly on histology [4]. Surgery is still the best therapeutic option with a generally good prognosis, especially in small-sized completely intra-thyroid DTC without lymph nodal or distant metastases [4,14]. Conversely, PDTC and ATC are more aggressive with a severe natural history. In these cases, there is usually no chance for curative surgery, only palliative procedures [25,26].

Between these two last extremes described, there are several intermediate conditions showing a locally advanced tumour that might benefit from a radical resection but need more complex procedures than total thyroidectomy. Indeed, considering the neck’s anatomy, when thyroid tumours acquire the capacity to infiltrate the neighbourhood, a radical resection is a difficult goal. In this regard, infiltration of the airway represents the most challenging condition, but, in a consistent number of patients, radical surgery can be performed and increases the chances for cure [8]. The shaving-off of the tumour from the airway, tracheal window resection or segmental resection are performed in relation to the degree of airway invasion [4]. These procedures, already described and still disputed in some technical aspects and indications [8,24], are effective in expert hands but must be strictly planned [27]. The preoperative US evaluation of thyroid nodules allows the identification of some characteristics, such as the higher tumour size or the irregular tumour margins, typically associated with an already locally advanced disease and more aggressive histotype [15]. However, although the US examination aims at also assessing lymph node status, it might fail to adequately investigate the deep neck. Furthermore, the cytological information derived from FNAs cannot currently predict the biological aggressiveness of well-differentiated carcinomas.

In particular, this lack of knowledge prevents us from gaining specific information on the tumour trend to present local infiltration, early lymphatic spread or both. To fill this gap, we attempted to investigate the IHC expression of AhR, N-cadherin, E-cadherin and CD147, considering the correlation found in various cancer types between their expression and tumour aggressive behaviour [16,17,18,19,20,21,22,23].

Indeed, based on the study findings, we could advance the potential role of AhR/N-cadherin as a prognostic marker, being able to suggest a more aggressive phenotype in thyroid carcinoma as evidenced by the high levels of AhR/N-cadherin found in most patients of the PTC-A and PDTC/ATC groups.

These findings are consistent with previously reported data that showed how the increased expression of AhR may play an oncogenic enhancing function not only through the induction of an immune-tolerant microenvironment but also through the expression of proteins such as N-cadherin involved in the regulation and initiation of the EMT [17,28,29].

In our case series, AhR seems to be an important prognostic marker, highly expressed in patients with a persistence or recurrence of disease in addition to being a marker of aggression and histologic de-differentiation.

N-cadherin expression still remains a matter of debate in the context of thyroid oncogenesis [17,30]. This study could find that expression of this protein plays a significant role in promoting invasiveness, being expressed in both PTC-A and PDTC/ATC groups and displaying a low expression rate in the PTC-B group.

The role of E-cadherin and CD147 remains to be defined: despite the available data reported [31,32,33,34,35,36], they showed only marginal roles as markers of aggressive biological behaviour in this analysis. In particular, although E-cadherin expression is not significantly associated with all of the analysed variables, we could observe a trend of increased aggressiveness (PTC-A and PDTC/ATC) with absent or very low E-cadherin. CD147 was highly expressed both in PTC-B and PDTC/ATC groups and increased with a decrease in E-cadherin. Nevertheless, as CD147 levels increased, both AhR and N-cadherin expressions decreased; some authors hypothesised that the reason behind the biological aggressiveness related to the overexpression of CD147 could be the enhancement of the EMT process with cancer migration and invasion as also described in other tumours [37,38,39].

The current study has some limitations: this is a retrospective clinical study and poor a priori knowledge is available to formally attribute an effective correlation between AhR/N-cadherin expression and biologic aggressiveness. The study timeframe is almost two decades and does not guarantee homogeneous diagnostic–therapeutic management in all patients; moreover, the number of patients in group A is limited, despite it representing a good experience for a very rare condition, and, hence, it cannot fully show an independent impact of these findings. However, to have a more homogeneous caseload, the study was designed to only analyse PTCs among the most differentiated histotypes. In addition, considering that one of the goals of the study was to preoperatively identify non-infiltrating airway carcinomas and infiltrating ones, we considered only PTCs, since the preoperative FNAs do not allow the cytological diagnosis of follicular thyroid carcinomas. Therefore, the assessment of the analysed biomarkers on such specimens would not change the surgical management of preoperative follicular lesions. Moreover, since BRAFV600E mutation was demonstrated to be associated with an increased expression of AhR, particularly at the infiltrative tumour edge, in thyroid cancer murine models [17], and the associations between the molecules here investigated and other mutations implied in thyroid cancer pathogenesis, progression and prognosis (e.g., RET/PTC, TP53, TERT, ATK1 and RAS) have not yet been described [40], additional studies at the molecular level of these aspects should be conducted.

This study suggests that knowing in advance the most important onco-biological factors might be helpful in the decision-making process, providing a more solid therapeutic indication and an increased expectation for radical surgery when markers of local aggressiveness are negative. Hence, there is the need for further studies to confirm these preliminary findings. When prognosticators and biomarkers for local aggressiveness can be translated into clinical practice, they may be supportive in better planning the most appropriate surgical procedure to be performed. A biological tumour profile could change the current paradigm to plan treatments based on “static” instrumental examination, giving a more “dynamic” assessment of tumour behaviour from the cytological immunophenotyping of the FNA-derived material.

In conclusion, thyroid cancer is a varied disease with a positive outcome after resection, if the tumour is intrathyroid. Our study suggests that several markers deserve to be further investigated in view of a potential role to discriminate, among the same histotype, between different subsets of a patient’s risk. Knowing in advance tumour aggressiveness could be helpful in avoiding suboptimal surgery if the tumour has an inner trend to recur or to infiltrate the neighbouring anatomical structures.

## Figures and Tables

**Table 1:** H-score cut-offs for N-cadherin, AhR and CD147 groups of expression.

	H-Scores for Expression Groups	
Protein (IHC)	Low	High
N-Cadherin	0–40	41–300
AhR	0–70	71–300
CD147	0–40	41–300

**Table 2:** Clinical characteristics of patients with PTC invading the airway.

ID	Sex	Age	Pathological Diagnosis	Extrathyroidal Invasion	Multifocality	Tumour Size (cm)	pT	Lymph Node Metastasis	N Tot	N+	AJCC Stage at Diagnosis	Recurrence or Persistence
1	F	69	PTC	yes	yes	4.0	pT3	yes	5	5	II	-
2	F	75	PTC	yes	yes	2.4	pT4	no	10	0	III	Persistence
3	F	59	PTC	yes	yes	1.8	pT3	yes	1	1	II	Recurrence
4	F	45	PTC	yes	no	1.8	pT4	no	0	0	III	Recurrence
5	M	49	PTC	yes	no	2.4	pT3	no	3	0	II	Persistence
6	F	72	PTC	yes	no	2.0	pT4	yes	11	2	III	Persistence
7	F	55	PTC	yes	no	1.2	pT4	no	0	0	III	Persistence
8	M	43	PTC	yes	yes	2.2	pT4	yes	5	5	I	Recurrence

**Table 3:** N-cadherin, AhR and CD147 expression among tumour groups.

Histotype	N-Cadherin			AhR			CD147		
	Low	High	*p*	Low	High	*p*	Low	High	*p*
	n. (%)	n. (%)		n. (%)	n. (%)		n. (%)	n. (%)	
PTC-A ^1^	1 (13)	7 (87)		4 (50)	4 (50)		5 (63)	3 (37)	
PTC-B ^2^	20 (74)	7 (26)	0.005	18 (67)	9 (33)	<0.001	4 (15)	23 (85)	0.011
PDTC/ATC	9 (45)	11 (55)		1 (5)	19 (95)		3 (15)	17 (85)	

^1^ PTC-A: PTC infiltrating the airway. ^2^ PTC-B: PTC without airway infiltration.

**Table 4:** E-cadherin distribution among tumour groups.

Histotype	E-Cadherin		
	Normal	Lost/Low	*p*
	n. (%)	n. (%)	
PTC-A ^1^	0 (0)	8 (100)	
PTC-B ^2^	3 (11)	24 (89)	0.516
PDTC/ATC	3 (15)	17 (85)	

^1^ PTC-A: PTC infiltrating the airway. ^2^ PTC-B: PTC without airway infiltration.

**Table 5:** Expression of N-cadherin, AhR, CD147 or E-cadherin associations with clinical–pathological characteristics.

Parameter	N-Cadherin			AhR			CD147			E-Cadherin			Total
	Low	High	*p*	Low	High	*p*	Low	High	*p*	N ^1^	L/L ^2^	*p*	
	n. (%)	n. (%)		n. (%)	n. (%)		n. (%)	n. (%)		n. (%)	n. (%)		n. (%)
Histotype													50 (91)
PTC	21 (60)	14 (40)	0.193	22 (63)	13 (37)	<0.001	9 (26)	26 (74)	0.910	3 (9)	32 (91)	0.607	35 (70)
PDTC/ATC	6 (40)	9 (60)		1 (7)	14 (93)		3 (20)	12 (80)		2 (13)	13 (87)		15 (30)
Multifocality													38 (69)
No	11 (55)	9 (45)	0.973	9 (45)	11 (55)	0.090	4 (20)	16 (80)	0.573	1 (5)	19 (95)	0.485	20 (53)
Yes	10 (56)	8 (44)		13 (72)	5 (28)		5 (28)	13 (72)		2 (11)	16 (89)		18 (47)
Nodal Status													36 (65)
N−	9 (60)	6 (40)	0.864	7 (47)	8 (53)	0.230	1 (7)	14 (93)	0.058	1 (7)	14 (93)	0.760	15 (42)
N+	12 (57)	9 (43)		14 (67)	7 (33)		7 (33)	14 (67)		2 (10)	19 (90)		21 (58)
Recurrence/Persistence													45 (82)
No	12 (60)	8 (40)	0.182	12 (60)	8 (40)	0.031	4 (20)	16 (80)	0.366	1 (5)	19 (95)	0.243	20 (44)
Yes	10 (40)	15 (60)		7 (28)	18 (72)		8 (32)	17 (68)		4 (16)	21 (84)		25 (56)

^1^ N: normal expression. ^2^ L/L: lost/low expression.

**Table 6:** Associations between tumours examined according to the concurrent expression of AhR and N-cadherin and clinical–pathological parameters.

Parameter	AhR and N-Cadherin			Total
	Low	High	*p*	
	n. (%)	n. (%)		n. (%)
Histotype				30 (100)
PTC	14 (70)	6 (30)	0.002	20 (67)
PDTC/ATC	1 (10)	9 (90)		10 (33)
Multifocality				23 (100)
No	5 (50)	5 (50)	0.417	10 (43)
Yes	9 (31)	4 (69)		13 (57)
Nodal Status				22 (100)
N−	6 (55)	5 (45)	0.659	11 (50)
N+	8 (73)	3 (27)		11 (50)
Recurrence/Persistence				26 (100)
No	7 (70)	3 (30)	0.043	10 (38)
Yes	4 (25)	12 (75)		16 (62)

**Table 7:** PTC-A and PTC-B associations with immunomarkers.

Parameter	PTC			Total
	PTC-A ^1^	PTC-B ^2^	*p*	
	n. (%)	n. (%)		n. (%)
	8 (23)	27 (77)		35 (100)
N-cadherin				
Low	1 (5)	20 (95)	0.003	21 (60)
High	7 (50)	7 (50)		14 (40)
AhR				
Low	4 (18)	18 (82)	0.433	22 (63)
High	4 (31)	9 (69)		13 (37)
CD147				
Low	5 (56)	4 (44)	0.015	9 (26)
High	3 (12)	23 (88)		26 (74)
E-cadherin				
N ^3^	0 (0)	3 (100)	1.000	3 (9)
L/L ^4^	8 (25)	24 (75)		32 (91)

^1^ PTC-A: PTC infiltrating the airway. ^2^ PTC-B: PTC without airway infiltration. ^3^ N: normal expression. ^4^ L/L: lost/low expression.

## Supplementary Materials

The following are available online at https://www.mdpi.com/article/10.3390/jcm10194351/s1. Figure S1: (a) Negative correlation between the expression of CD147, E-cadherin and (b) AhR. Figure S2: positive correlation between the expression of AhR and E-cadherin.
