# Supplementary material for: Impact of Epithelial–Mesenchymal Immunophenotype on Local Aggressiveness in Papillary Thyroid Carcinoma Invading the Airway
Source: J Clin Med. 2021 Sep 24;10(19):4351. doi: 10.3390/jcm10194351 (PMC8509765; doi:10.3390/jcm10194351)
Supplement: Supplementary file 1 [file jcm-10-04351-s001.zip › jcm-1326470-supplementary.pdf]

Supplementary Materials

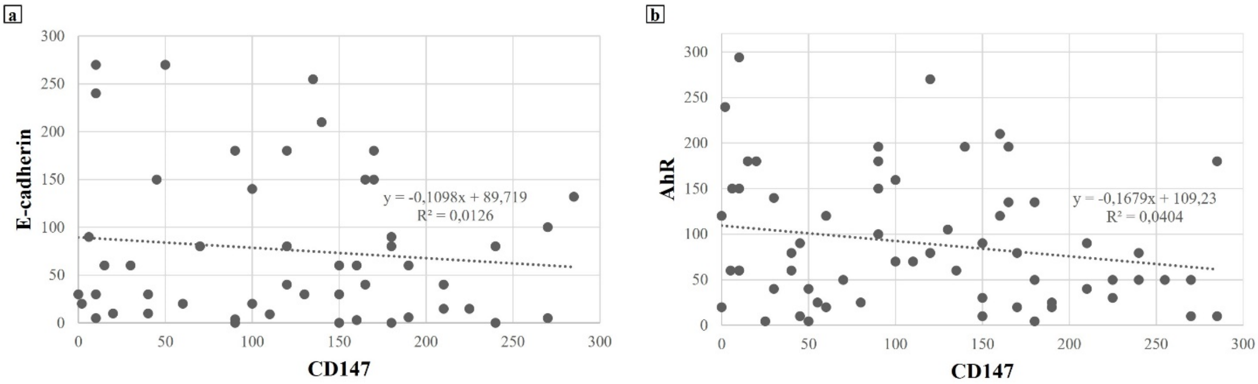


Figure S1. (a) Negative correlation between the expression of CD147, E-cadherin and (b) AhR.

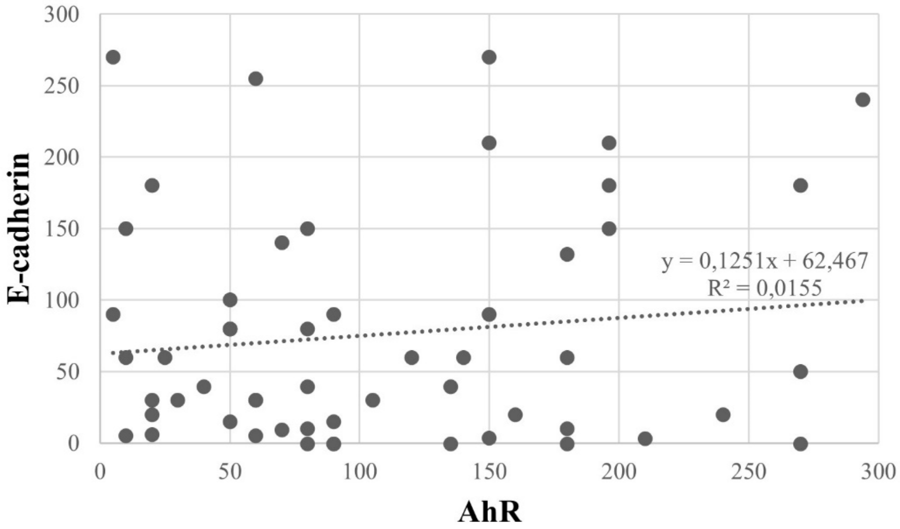


Figure S2. Positive correlation between the expression of AhR and E-cadherin.
